# The Weekend Effect on Outcomes for Type A Aortic Dissection Surgery: A Nationwide Analysis

**DOI:** 10.1093/icvts/ivag067

**Published:** 2026-03-02

**Authors:** Christopher M Van Hove, Samuel R Harris, Erik Beckmann, Jesse M Manunga, Michael S Megaly, Michael D Miedema, Evan Walser-Kuntz, Kevin M Harris

**Affiliations:** Minneapolis Heart Institute at Abbott Northwestern Hospital, Minneapolis, MN 55407, United States; College of Engineering and Applied Science, University of Colorado, Boulder, CO 80309, United States; Minneapolis Heart Institute Foundation at Abbott Northwestern Hospital, Minneapolis, MN 55407, United States; Minneapolis Heart Institute Foundation at Abbott Northwestern Hospital, Minneapolis, MN 55407, United States; Ascension St. John Medical Center, Tulsa, OK 74104, United States; Minneapolis Heart Institute Foundation at Abbott Northwestern Hospital, Minneapolis, MN 55407, United States; Minneapolis Heart Institute Foundation at Abbott Northwestern Hospital, Minneapolis, MN 55407, United States; Minneapolis Heart Institute Foundation at Abbott Northwestern Hospital, Minneapolis, MN 55407, United States

**Keywords:** aortic dissection, surgery, weekend

## Abstract

**Objectives:**

Weekend admission has been shown to influence outcomes with surgical and medical conditions. This study aimed to evaluate if surgical outcomes for acute type A aortic dissection (TAAD) vary based on weekday or weekend admission.

**Methods:**

The Nationwide Readmissions Database was queried for hospitalizations with TAAD as a primary diagnosis that underwent surgical procedures over a 5-year period. The association between TAAD and outcomes (in-hospital mortality, days to procedure, length of stay, hospital costs, and re-admission) based on day of admission (weekday versus weekend) was examined.

**Results:**

A total of 14 260 patients admitted to the hospital with TAAD (10 582 [74.2%] weekdays and 3678 [25.8%] weekends) were included in this analysis. Patients admitted on the weekdays tended to be older, and more commonly had undergone prior cardiac surgery. Neurologic, coronary, and renal malperfusion were more common in patients admitted on the weekend. Compared to weekday admissions with aortic dissection, weekend admissions were associated with higher in-hospital mortality (17.3% vs 15.4%, *P* = .027) and also associated with increased risk in a multivariate analysis. Large hospital size and teaching status also had more favourable outcomes in the multivariate analysis.

**Conclusions:**

While known aortic dissection risk factors significantly impact TAAD survival, patients admitted on weekends also demonstrate a higher mortality rate.

## Graphical abstract

**Figure:**
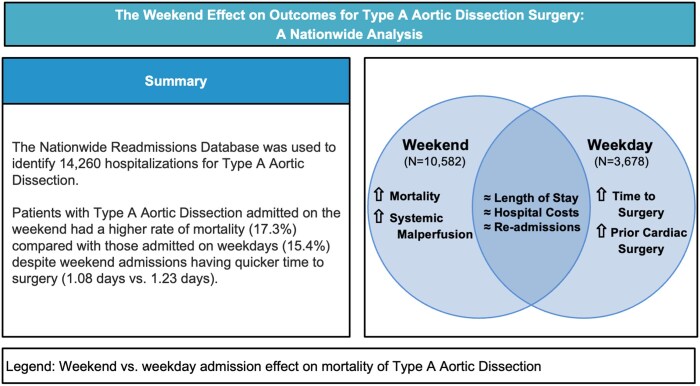

## INTRODUCTION

Acute type A aortic dissection (TAAD) requires early recognition and surgical treatment with patients benefitting from surgical expertise and multidisciplinary coordination of care.^1^ Prior studies of a variety of acute vascular conditions including acute coronary syndrome, pulmonary embolism, and abdominal aortic aneurysm have shown increased mortality and prolonged length of stay for patients admitted on weekends compared to those admitted on weekdays.^2–7^ The reasons for this weekend effect are felt to relate to staffing on the weekends as well as the level of expertise that may be available.^2^^,^^3^ There is ongoing debate if TAAD outcomes are influenced by the day of admission. Thus, in this investigation, the impact of admission day on mortality is evaluated as well as length of stay, costs, and re-admission.

## METHODS

The Nationwide Readmissions Database (NRD) is the largest database for inpatient re-admissions from all payers in the United States with approximately 35 million discharges annually. It is part of the Healthcare Cost and Utilization Project (HCUP) that is sponsored by Agency for Healthcare Research and Quality. This database captures hospital stays as individual observations with variables including diagnoses, procedures, discharges, and outcomes among others.^4^ Data from the NRD are deidentified and publicly available, and hence, the present study was exempted from Institutional Board Review oversight. We identified our population based on the primary diagnosis (International Classification of Diseases, Tenth Revision, Clinical Modification codes I71.0x) equivalent to the same group (International Classification of Diseases, ninth Revision, Clinical Modification codes 44101) used in prior studies (**Table S1**).^5^^,^^6^ The study cohort was restricted to patients with urgent admissions who underwent surgery for TAAD and were identified using corresponding procedural codes (**Table S2**) for ascending aorta/arch dissection repair, consistent with the design of recent studies.^5^^,^^7^ Weekday and weekend admissions were identified from the HCUP-NRD database entries between October 2016 and December 2021. Weekend admission is defined as a hospital admission occurring on a Saturday or a Sunday based on NRD predefined calendar days which are recorded from midnight to midnight. Patient characteristics (including age and gender), pre-op complications, hospital characteristics including hospital size (large, medium, and small as defined by using a composite of geographical location, teaching status, and number of beds), location (rural is defined as a community size <50 000 people based on the National Center for Health Statistics), and teaching status (defined by meeting 1 of 3 criteria: membership in the Association of American Medical Colleges recognized Council of Teaching Hospitals, the presence of a residency programme approved by the Accreditation Council for Graduate Medical Education, or the ratio of full-time equivalent interns and residents greater than or equal to 0.25) were evaluated. Patients transferred after admission were only classified by the final destination hospital. The primary outcome was all cause mortality during the same hospital stay as recorded in the database. Secondary outcomes include length of stay and hospital costs. Urgent re-admission, defined as a hospitalization within 30 days of discharge from the index hospitalization, was evaluated in patients admitted before December in order to evaluate patients re-admitted in the same calendar year. As the dataset overlapped with the COVID-19 pandemic, a sensitivity analysis was performed examining time to surgery, length of stay, and mortality before and after March 2020. Categorical data were expressed as count (%), while continuous data were expressed as mean (+/- standard deviation). Baseline demographics and perioperative complications were compared using Chi-square test for categorical variables and Mann-Whitney *U*-test for continuous variables. Multivariable quasi-Poisson regression was used to estimate relative risk with corresponding 95% confidence interval for the primary and secondary outcomes as well as to assess for other risk factors incorporated in the regression model. Covariates selected for the multivariable analysis include age, sex, and prior cardiac surgery, a known risk factor for hospital mortality,^1^ as well as variables found to have *P* < .1 in simple linear regression. All tests were 2-tailed and considered statistically significant with *P* value <.05. All statistical analysis was performed using R version 4.2.2.

## RESULTS

As presented in **Table 1**, a total of 14 260 hospitalizations were examined (10 582 [74.2%] weekdays and 3678 [25.8%] weekends). A majority of patients were male (65.8%) with a mean age of 70.7 ± 13.7 years. Patients admitted on the weekend were younger (weekend 60.3 ± 13.5, weekday 60.9 ± 13.7, *P* = .034) and more likely to have Medicaid and conversely less likely to have Medicare insurance. Hospital characteristics, such as size, location, and teaching status, were comparable for both patient groups.

**Table 1.** Characteristics of the Study Population

	Total (*n* = 14 260)	Weekday (*n* = 10 582)	Weekend (*N* = 3678)	*P* value
Sex				.631
Male	9381 (65.79%)	6949 (65.67%)	2432 (66.12%)	
Female	4879 (34.21%)	3633 (34.33%)	1246 (33.88%)	
Age				.013
< 70 years	10114 (70.93%)	7446 (70.36%)	2668 (72.54%)	
> 70 years	4146 (29.07%)	3136 (29.64%)	1010 (27.46%)	
Income				
First quartile	3602 (25.26%)	2639 (24.94%)	963 (26.18%)	.131
Second quartile	3554 (24.92%)	2665 (25.18%)	889 (24.17%)	.240
Third quartile	3471 (24.34%)	2600 (24.57%)	871 (23.68%)	.302
Fourth quartile	3446 (24.17%)	2542 (24.02%)	904 (24.58%)	.491
Primary payer status				
Medicare^a^	6036 (42.33%)	4556 (43.05%)	1480 (40.24%)	.003
Medicaid^b^	2167 (15.20%)	1567 (14.81%)	600 (16.31%)	.030
Private	4720 (33.10%)	3483 (32.91%)	1237 (33.63%)	.429
Self-pay	779 (5.46%)	582 (5.50%)	197 (5.36%)	.777
No charge	67 (0.47%)	40 (0.38%)	27 (0.73%)	.010
Other	460 (3.23%)	332 (3.14%)	128 (3.48%)	.336
Hospital characteristics				.301
Rural	4804 (33.69%)	3591 (33.93%)	1213 (32.98%)	
Urban	9456 (66.31%)	6991 (66.07%)	2465 (67.02%)	
Teaching status				.879
Non-teaching	1333 (9.35%)	992 (9.37%)	341 (9.27%)	
Teaching	12927 (90.65%)	9590 (90.63%)	3337 (90.73%)	
Bed size				
Small bed size	583 (4.09%)	438 (4.14%)	145 (3.94%)	.638
Medium bed size	2514 (17.63%)	1882 (17.78%)	632 (17.18%)	.424
Large bed size	11163 (78.28%)	8262 (78.08%)	2901(78.87%)	.323
Transfer	1446 (10.14%)	1104 (10.43%)	342 (9.30%)	.053
Comorbidities				
Marfan syndrome	237 (1.66%)	174 (1.64%)	63 (1.71%)	.837
AIDS	56 (0.39%)	39 (0.37%)	17 (0.46%)	.529
Alcohol use disorder	532 (3.73%)	387 (3.66%)	145 (3.94%)	.462
Lymphoma	47 (0.33%)	36 (0.34%)	11 (0.30%)	.835
Leukaemia	43 (0.30%)	36 (0.34%)	7 (0.19%)	.210
Metastatic cancer	58 (0.41%)	41 (0.39%)	17 (0.46%)	.643
Solid tumour without metastasis, malignant	205 (1.44%)	156 (1.47%)	49 (1.33%)	.587
Cerebrovascular disease	790 (5.54%)	574 (5.43%)	216 (5.87%)	.326
Heart failure	3405 (23.88%)	2554 (24.14%)	851 (23.14%)	.230
Dementia	24 (0.17%)	19 (0.18%)	5 (0.14%)	.747
Diabetes	1929 (13.53%)	1407 (13.30%)	522 (14.19%)	.18
Drug use disorder	723 (5.07%)	522 (4.93%)	201 (5.46%)	.221
Cocaine use	325 (2.28%)	230 (2.17%)	95 (2.58%)	.171
Hypertension	11852 (83.11%)	8786 (83.03%)	3066 (83.36%)	.661
Severe liver disease	89 (0.62%)	54 (0.51%)	35 (0.95%)	.005
Morbid obesity	1202 (8.43%)	875 (8.27%)	327 (8.89%)	.256
Paralysis	22 (0.15%)	13 (0.12%)	9 (0.24%)	.168
Peripheral vascular disease	2893 (20.29%)	2158 (20.39%)	735 (19.98%)	.611
Advanced renal failure	494 (3.46%)	381 (3.60%)	113 (3.07%)	.145
Hypothyroidism	1129 (7.92%)	864 (8.16%)	265 (7.21%)	.069
Valvular disease	3822 (26.80%)	2864 (27.06%)	958 (26.05%)	.238
Prior cardiac surgery	828 (5.81%)	647 (6.11%)	181 (4.92%)	.009
Methamphetamine use	225 (1.58%)	163 (1.54%)	62 (1.69%)	.594
ACS/MI	326 (2.29%)	230 (2.17%)	96 (2.61%)	.144
COPD	1867 (13.09%)	1371 (12.96%)	496 (13.49%)	.428
Renal malperfusion	7185 (50.39%)	5279 (49.89%)	1906 (51.82%)	.045
Mesenteric malperfusion	295 (2.07%)	219 (2.07%)	76 (2.07%)	1.000
Neurologic malperfusion	2429 (17.03%)	1761 (16.64%)	668 (18.16%)	.037
Coronary malperfusion	1086 (7.62%)	777 (7.34%)	309 (8.40%)	.040
Repair day				
Day 0	9114 (63.91%)	6691 (63.23%)	2423 (65.88%)	.004
Day 1	2783 (19.52%)	2078 (19.64%)	705 (19.17%)	.552
Day 2+	2363 (16.57%)	1813 (17.13%)	550 (14.95%)	.002

a Medicare is a federally subsidized, government-run health insurance providing coverage for those over the age of 65 and certain younger individuals with disability or serious medical conditions.

b Medicaid is a joint federal and state programme providing free or low-cost health insurance to people with low incomes. Abbreviations: ACS, acute coronary syndrome; AIDS, acquired immunodeficiency syndrome; COPD, chronic obstructive pulmonary disease; MI, myocardial infarction.

Most comorbidities (Marfan syndrome, heart failure, hypertension, diabetes mellitus, chronic kidney disease, obesity, cerebrovascular disease) were comparable across groups. Patients with weekend admissions were more likely to have severe liver disease and less likely to have a history of prior cardiac surgery (4.9% vs 6.1%, *P* = .009). Malperfusion of the renal, neurologic, and coronary distributions was slightly more common in the weekend patient group, though mesenteric malperfusion was similar in both groups.

As shown in **Table 2**, there were 663 (17.3%) in-hospital deaths among patients admitted on weekends compared to 1642 (15.4%) for patient admitted during the weekdays (*P* = .027). Patients admitted on the weekend were more likely to have surgery on the day of admission (days to procedure 1.08 vs 1.23, *P* < .001). Length of stay (14.9 vs 15.5 days, *P* = NS) and total hospital costs were similar between groups. The urgent re-admission rate was 18.1% and did not differ between weekend and weekday admission (18.4% vs 17.4%, *P* = NS). The mean time to readmission was 10.6 days. The most common reasons listed as primary diagnosis for readmission were cardiac (26.5%), aorta/vascular (19.9%), infectious (9.1%), bleeding (4.4%), and venous thromboembolism (3.6%).

**Table 2.** In-hospital Outcomes of Patients Based on Weekday Versus Weekend Admission

	Weekday	Weekend	*P* value
Days to procedure	1.23	1.08	<.001
Length of stay	15.54	14.85	.107
Cost	109227.2	1066698.5	.614
Re-admission	18.4%	17.4%	.248
Mortality	1692 (15.41%)	663 (17.33%)	.027

Multivariable regression revealed several variables associated with mortality as shown in **Table 3**. Older age was correlated with increased mortality. Differences were seen with income and insurance as a lower mortality was observed in those with higher income and with private insurance. Individuals at rural hospitals experienced higher mortality while patients at large hospitals and at teaching hospitals had lower mortality. Additionally, patients that required a transfer to another hospital had lower mortality. It was also observed that the weekend effect persisted for large hospitals (16.91% weekends vs 14.91% weekdays, *P* = .012), with a similar trend in teaching hospitals (16.62% weekend vs 15.18% weekday, *P* = .053).

**Table 3.** Patient Characteristics, Hospital Characteristics, and Patient Comorbidities in a Comparative Multivariable Analysis of Weekend Versus Weekday Admissions

	Relative risk	t value	*P* value
Female	1.06 (0.98, 1.15)	1.451	.147
Age	1.02 (1.01, 1.02)	8.878	<.001
Income (vs Q1)			
Second quartile	0.96 (0.86, 1.06)	−0.877	.380
Third quartile	0.88 (0.79, 0.98)	−2.42	.016
Fourth quartile	0.80 (0.72, 0.90)	−3.787	<.001
Primary payer status (vs Medicare)			
Medicaid	1.01 (0.88, 1.17)	0.187	.852
Private insurance	0.89 (0.80, 1.00)	−1.985	.047
Self-pay	1.41 (1.17, 1.68)	3.755	<.001
No charge	1.15 (0.62, 1.94)	0.492	.623
Other	1.00 (0.80, 1.24)	0.018	.986
Rural	1.24 (1.14, 1.34)	5.178	<.001
Teaching status	0.85 (0.76, 0.96)	−2.656	.008
Bed size (vs small)			
Medium	0.96 (0.80, 1.16)	−0.434	.664
Large	0.83 (0.70, 0.99)	−2.158	.031
Marfan syndrome	0.84 (0.55, 1.21)	−0.892	.372
Alcohol use disorder	0.96 (0.77, 1.19)	−0.362	.718
Lymphoma	0.26 (0.05, 0.74)	−2.094	.036
Cerebrovascular disease	1.12 (0.96, 1.30)	1.419	.156
Heart failure	1.37 (1.26, 1.49)	7.321	<.001
Cocaine use	0.95 (0.67, 1.33)	−0.293	.769
Drug use disorder	0.88 (0.69, 1.10)	−1.098	.272
Hypertension	0.65 (0.60, 0.71)	−9.353	<.001
Severe liver disease	2.25 (1.68, 2.94)	5.711	<.001
Paralysis	1.97 (0.93, 3.60)	1.98	.048
Peripheral vascular disease	1.06 (0.96, 1.16)	1.174	.240
Valvular disease	0.92 (0.84, 1.00)	−1.956	.050
ACS/MI	1.32 (1.07, 1.61)	2.651	.008
Prior cardiac surgery	1.16 (0.99, 1.35)	1.844	.065
COPD	1.02 (0.92, 1.14)	0.404	.686
Transfer	0.73 (0.63, 0.83)	−4.585	<.001
Renal malperfusion	1.40 (1.29, 1.51)	8.148	<.001
Mesenteric malperfusion	2.80 (2.38, 3.28)	12.596	<.001
Neurologic malperfusion	1.47 (1.35, 1.60)	8.592	<.001
Coronary malperfusion	1.65 (1.45, 1.88)	7.576	<.001
Weekend admission	1.09 (1.00, 1.19)	2.081	.041

Abbreviations: ACS, acute coronary syndrome; COPD, chronic obstructive pulmonary disease; MI, myocardial infarction.

Medical comorbidities that demonstrated higher mortality included heart failure, severe liver disease, paralysis, and prior myocardial infarction. Malperfusion was associated with significant risk whether it involved renal, mesenteric, neurologic or coronary beds. In the multivariable analysis, weekend admit was associated with increased mortality (RR 1.09, *P* = .04).

To evaluate the impact of the COVID-19 pandemic, key parameters were compared before and after its onset. Mean time to surgery (1.19 vs 1.19 days, *P* = .926), length of stay (14.69 vs 15.58 days, *P* = .203), and mortality (15.83% vs 16.09%, *P* = .689) did not differ for the cohort as a whole. Similarly, when the analysis was restricted to weekend admissions, mortality remained comparable (15.5% vs 17.75%, *P* = .428).

## DISCUSSION

In this large study of over 14 000 patients utilizing a nationwide database, the impact of admission day on in-hospital mortality and resource utilization was evaluated. Patients admitted on the weekend tended to be younger, with less prior cardiac surgery but more likely to have renal, neurologic, and coronary malperfusion. While typical aortic dissection clinical risk factors were associated with increased mortality in multivariate analysis, weekend admit was also associated with increased mortality.

The reasons for increased in-hospital mortality for weekend admissions of TAAD are not totally clear. It is known that hospitals have fewer physicians (including surgeons, cardiologists, radiologists, and anesthesiologists), nurses, and support staff available on weekends. Given the life-threatening nature of TAAD, prompt diagnosis and initial management of these patients is critical. Despite the potential for reduced staffing, time to surgery was shorter on weekends, likely reflecting greater surgeon and operating room availability in the absence of elective cases. However, mortality remained higher on weekends despite this time advantage, suggesting that other factors including limited support staff may offset the benefits of earlier operative intervention.^2^^,^^3^^,^^8–11^

The impact of surgeon volume on outcomes of patients with acute aortic pathologies cannot be underestimated. It is recognized that in patients undergoing aortic aneurysm repair, improved outcomes were seen in patients with ruptured aneurysms treated using open repair by high-volume surgeons compared to low volume surgeons.^12^ Similar results have also been seen with improved outcomes associated with both higher institutional and surgeon case volume for TAAD.^13^ An increase in mortality has previously been seen in patients with ruptured thoracic and abdominal aortic aneurysms admitted over the weekend compared to those admitted during a weekday.^10^ The availability of a high-volume surgeon over the weekend may be limited and with call coverage of other specialties, the surgeon may not be working with the multidisciplinary team they are most accustomed to working with.

There are limited North American data and overall contradictory data regarding the weekend effect in TAAD. A prior study from early 2000s in Italy showed a higher mortality (OR 1.31 *P* < .001) for acute aortic dissection (AD) patients admitted on the weekend.^14^ In a study from the United States from an earlier time frame (2004-2012), a higher mortality (adjusted OR 1.17, *P* = .002) was seen on weekend admissions with AD as well as decreased use of interventions and lower proportion of patients who underwent aortic repair early during hospital stay.^5^ Similarly, weekend presentation was associated with 13% increased mortality (OR 1.17, *P* = .003) in a study utilizing the National Emergency Department Sample.^15^ In contrast, weekend admission was not associated with mortality in other studies.^16–20^ Of note, analysis looking at night versus daytime surgical repair of type A aortic dissection have not demonstrated a mortality difference.^17^^,^^18^^,^^20^^,^^21^ Recently, Takahashi et al^22^ evaluated the effects of an inpatient transfer system in the Tokyo metropolitan area. They found that after the establishment of a transfer system, the higher mortality for weekend admissions was no longer observed.

This study does confirm in multivariate analysis numerous well-established predictors of mortality for aortic dissection. These include clinical variables including advanced age and malperfusion of any vascular distribution including myocardial ischaemia.^1^ Additionally, higher income and private insurance were also associated with improved outcomes.^6^^,^^7^

Hospital variables including larger hospital size, transfer, and teaching status play a role in better outcomes as has been shown previously.^23^ Transfer to high-volume hospitals may have favourable benefits for surgical treatment of TAAD.^24^ Since publication of some of the earlier studies evaluating the weekend effect, there has been an emphasis on dedicated aortic centres and systems of care for acute TAAD.^18^^,^^22^^,^^25–27^ In fact, Takahashi et al’s^22^ study suggests that the development of a transfer system can obliterate the prior observed weekend effect for TAAD. Thus, there is a potential favourable impact of multidisciplinary aortic care systems for patients with complex aortic disease and deserving of further study regarding the potential impact after hours.^18^^,^^22^^,^^25–27^

As this is a retrospective study using an administrative database with non-randomized treatment assignment, there is potential for selection bias (due to miscoding) and residual confounding variables to influence the results, despite multivariable adjustment.^5^^,^^28^ In addition, because patients were identified based on TAAD procedure codes to mitigate misclassification associated with reliance on specific ICD-10 diagnosis codes alone, this study was unable to distinguish medically managed TAAD patients from those with Type B aortic dissection.

As with other large administrative databases, the NRD lacks granularity across several predefined variables. For instance, temporal precision is limited as time is recorded by hospital day rather than by hours, which may partially account for the number of patients undergoing surgery on day 2. Similarly, the timing of admission was not available to compare differences across daytime versus nighttime admissions, although recent studies demonstrated no significant difference in time of day on mortality amongst patients with TAAD.^17^^,^^18^^,^^20^^,^^21^ Since this study focused on patients undergoing surgery, as with other acute dissection studies, we lack data on patients that may have suffered acute TAAD but died either before presentation or sometime after presentation enroute for surgery at the same or another centre.

Lastly, it is important to note that this study’s findings are limited to in-hospital events and re-admission. Although secondary end-points may help contextualize the primary end-point of in-hospital mortality, all secondary analyses should be interpreted as exploratory.

## CONCLUSIONS

The increased mortality for acute TAAD patients admitted on weekends seen in this study raises questions regarding the optimal way to provide uniform care off hours. Efforts should be made to further examine clinician and systems specific improvements to benefit the care of all AD patients regardless of the timing of admission.

## Supplementary Material

ivag067_Supplementary_Data

## Data Availability

The data underlying this article are available in the article and in its online supplementary material.

## ABBREVIATIONS

HCUP: Healthcare Cost and Utilization Project
NRD: National Readmission Database
TAAD: acute type A aortic dissection
