# Supplementary material for: The Weekend Effect on Outcomes for Type A Aortic Dissection Surgery: A Nationwide Analysis
Source: Interdiscip Cardiovasc Thorac Surg. 2026 Mar 2;41(3):ivag067. doi: 10.1093/icvts/ivag067 (PMC12987766; doi:10.1093/icvts/ivag067)
Supplement: ivag067_Supplementary_Data [file ivag067_supplementary_data.docx]

|  | ICD-10 Code |
| --- | --- |
| Marfan syndrome | Q87.4, Q87.40, Q87.41, Q87.42, Q87.43, Q87.44, Q87.45, Q87.46, Q87.47, Q87.48, Q87.49 |
| HIV/AIDS | B20, B20.0, B20.1, B20.2, B20.3, B20.4, B20.5, B20.6, B20.7, B20.8, B20.9, B21.0, B21.1, B21.2, B21.3, B22.0, B22.1, B22.8, B23.0, B23.1, B23.8, B24.0, B24.1, B24.8, Z21, B21, B22, B23, B24 |
| Alcohol use disorder | F10.1, F10.10, F10.11, F10.12, F10.13, F10.14, F10.15, F10.16, F10.17, F10.2, F10.20, F10.21, F10.22, F10.23, F10.24, F10.25, F10.26, F10.27, F10.9, F10.90 |
| Lymphoma | C81.00, C81.01, C81.02, C81.03, C81.04, C81.05, C81.06, C81.07, C81.08, C81.09, C81.10, C81.11, C81.12, C81.13, C81.14, C81.15, C81.16, C81.17, C81.18, C81.19, C81.20, C81.21, C81.22, C81.23, C81.24, C81.25, C81.26, C81.27, C81.28, C81.29, C81.30, C81.31, C81.32, C81.33, C81.34, C81.35, C81.36, C81.37, C81.38, C81.39, C81.40, C81.41, C81.42, C81.43, C81.44, C81.45, C81.46, C81.47, C81.48, C81.49, C81.70, C81.71, C81.72, C81.73, C81.74, C81.75, C81.76, C81.77, C81.78, C81.79, C81.90, C81.91, C81.92, C81.93, C81.94, C81.95, C81.96, C81.97, C81.98, C81.99, C82.00, C82.01, C82.02, C82.03, C82.04, C82.05, C82.06, C82.07, C82.08, C82.09, C82.10, C82.11, C82.12, C82.13, C82.14, C82.15, C82.16, C82.17, C82.18, C82.19, C82.20, C82.21, C82.22, C82.23, C82.24, C82.25, C82.26, C82.27, C82.28, C82.29, C82.30, C82.31, C82.32, C82.33, C82.34, C82.35, C82.36, C82.37, C82.38, C82.39, C82.40, C82.41, C82.42, C82.43, C82.44, C82.45, C82.46, C82.47, C82.48, C82.49, C82.50, C82.51, C82.52, C82.53, C82.54, C82.55, C82.56, C82.57, C82.58, C82.59, C82.60, C82.61, C82.62, C82.63, C82.64, C82.65, C82.66, C82.67, C82.68, C82.69, C82.80, C82.81, C82.82, C82.83, C82.84, C82.85, C82.86, C82.87, C82.88, C82.89, C82.90, C82.91, C82.92, C82.93, C82.94, C82.95, C82.96, C82.97, C82.98, C82.99, C83.00, C83.01, C83.02, C83.03, C83.04, C83.05, C83.06, C83.07, C83.08, C83.09, C83.10, C83.11, C83.12, C83.13, C83.14, C83.15, C83.16, C83.17, C83.18, C83.19, C83.30, C83.31, C83.32, C83.33, C83.34, C83.35, C83.36, C83.37, C83.38, C83.39, C83.50, C83.51, C83.52, C83.53, C83.54, C83.55, C83.56, C83.57, C83.58, C83.59, C83.70, C83.71, C83.72, C83.73, C83.74, C83.75, C83.76, C83.77, C83.78, C83.79, C83.80, C83.81, C83.82, C83.83, C83.84, C83.85, C83.86, C83.87, C83.88, C83.89, C83.90, C83.91, C83.92, C83.93, C83.94, C83.95, C83.96, C83.97, C83.98, C83.99, C84.00, C84.01, C84.02, C84.03, C84.04, C84.05, C84.06, C84.07, C84.08, C84.09, C84.10, C84.11, C84.12, C84.13, C84.14, C84.15, C84.16, C84.17, C84.18, C84.19, C84.40, C84.41, C84.42, C84.43, C84.44, C84.45, C84.46, C84.47, C84.48, C84.49, C84.60, C84.61, C84.62, C84.63, C84.64, C84.65, C84.66, C84.67, C84.68, C84.69, C84.70, C84.71, C84.72, C84.73, C84.74, C84.75, C84.76, C84.77, C84.78, C84.79, C84.90, C84.91, C84.92, C84.93, C84.94, C84.95, C84.96, C84.97, C84.98, C84.99, C84.A0, C84.A1, C84.A2, C84.A3, C84.A4, C84.A5, C84.A6, C84.A7, C84.A8, C84.A9, C84.Z0, C84.Z1, C84.Z2, C84.Z3, C84.Z4, C84.Z5, C84.Z6, C84.Z7, C84.Z8, C84.Z9, C85.10, C85.11, C85.12, C85.13, C85.14, C85.15, C85.16, C85.17, C85.18, C85.19, C85.20, C85.21, C85.22, C85.23, C85.24, C85.25, C85.26, C85.27, C85.28, C85.29, C85.80, C85.81, C85.82, C85.83, C85.84, C85.85, C85.86, C85.87, C85.88, C85.89, C85.90, C85.91, C85.92, C85.93, C85.94, C85.95, C85.96, C85.97, C85.98, C85.99, C86.0, C86.1, C86.2, C86.3, C86.4, C86.5, C86.6, C88.0, C88.2, C88.3, C88.4, C88.8, C88.9, C91.40, C91.41, C91.42, C91.43, C91.44, C91.45, C91.46, C91.47, C91.48, C91.49 |
| Leukemia | C90.00, C90.01, C90.02, C90.10, C90.11, C90.12, C90.20, C90.21, C90.22, C90.30, C90.31, C90.32, C91.00, C91.01, C91.02, C91.10, C91.11, C91.12, C91.30, C91.31, C91.32, C91.50, C91.51, C91.52, C91.60, C91.61, C91.62, C91.90, C91.91, C91.92, C92.00, C92.01, C92.02, C92.10, C92.11, C92.12, C92.20, C92.21, C92.22, C92.30, C92.31, C92.32, C92.40, C92.41, C92.42, C92.50, C92.51, C92.52, C92.60, C92.61, C92.62, C92.90, C92.91, C92.92, C93.00, C93.01, C93.02, C93.10, C93.11, C93.12, C93.30, C93.31, C93.32, C93.90, C93.91, C93.92, C94.00, C94.01, C94.02, C94.20, C94.21, C94.22, C94.30, C94.31, C94.32, C94.40, C94.41, C94.42, C94.6, C94.80, C94.81, C94.82, C95.00, C95.01, C95.02, C95.10, C95.11, C95.12, C95.90, C95.91, C95.92 |
| Metastatic cancer | C77.0, C77.1, C77.2, C77.3, C77.4, C77.5, C77.8, C77.9, C78.00, C78.01, C78.02, C78.1, C78.2, C78.30, C78.39, C78.4, C78.5, C78.6, C78.7, C78.80, C78.89, C79.00, C79.01, C79.02, C79.10, C79.11, C79.19, C79.2, C79.31, C79.32, C79.40, C79.49, C79.51, C79.52, C79.60, C79.61, C79.62, C79.70, C79.71, C79.72, C79.81, C79.82, C79.89, C79.9, C80.0 |
| Solid tumor without metastasis, malignant | C00.0, C00.1, C00.2, C00.3, C00.4, C00.5, C00.6, C00.8, C00.9, C01, C02.0, C02.1, C02.2, C02.3, C02.4, C02.8, C02.9, C03.0, C03.1, C03.9, C04.0, C04.1, C04.8, C04.9, C05.0, C05.1, C05.2, C05.8, C05.9, C06.0, C06.1, C06.2, C06.80, C06.89, C06.9, C07, C08.0, C08.1, C08.9, C09.0, C09.1, C09.8, C09.9, C10.0, C10.1, C10.2, C10.3, C10.4, C10.8, C10.9, C11.0, C11.1, C11.2, C11.3, C11.8, C11.9, C12, C13.0, C13.1, C13.2, C13.8, C13.9, C14.0, C14.2, C14.8, C15.3, C15.4, C15.5, C15.8, C15.9, C16.0, C16.1, C16.2, C16.3, C16.4, C16.5, C16.6, C16.8, C16.9, C17.0, C17.1, C17.2, C17.3, C17.8, C17.9, C18.0, C18.1, C18.2, C18.3, C18.4, C18.5, C18.6, C18.7, C18.8, C18.9, C19, C20, C21.0, C21.1, C21.2, C21.8, C22.0, C22.2, C22.3, C22.4, C22.7, C22.8, C22.9, C23, C24.0, C24.1, C24.8, C24.9, C25.0, C25.1, C25.2, C25.3, C25.4, C25.7, C25.8, C25.9, C30.0, C30.1, C31.0, C31.1, C31.2, C31.3, C31.8, C31.9, C32.0, C32.1, C32.2, C32.3, C32.8, C32.9, C33, C34.00, C34.01, C34.02, C34.10, C34.11, C34.12, C34.2, C34.30, C34.31, C34.32, C34.80, C34.81, C34.82, C34.90, C34.91, C34.92, C37, C38.1, C38.2, C38.3, C38.4, C38.8, C40.00, C40.01, C40.02, C40.10, C40.11, C40.12, C40.20, C40.21, C40.22, C40.30, C40.31, C40.32, C40.80, C40.81, C40.82, C40.90, C40.91, C40.92, C41.0, C41.1, C41.2, C41.3, C41.4, C41.9, C43.0, C43.10, C43.11, C43.12, C43.20, C43.21, C43.22, C43.30, C43.31, C43.39, C43.4, C43.51, C43.52, C43.59, C43.60, C43.61, C43.62, C43.70, C43.71, C43.72, C43.8, C43.9, C44.00, C44.01, C44.02, C44.09, C44.101, C44.1021, C44.1022, C44.109, C44.111, C44.1121, C44.1122, C44.119, C44.121, C44.1221, C44.1222, C44.129, C44.191, C44.1921, C44.1922, C44.199, C44.201, C44.202, C44.209, C44.211, C44.212, C44.219, C44.221, C44.222, C44.229, C44.291, C44.292, C44.299, C44.300, C44.301, C44.309, C44.310, C44.311, C44.319, C44.320, C44.321, C44.329, C44.390, C44.391, C44.399, C44.40, C44.41, C44.49, C44.500, C44.501, C44.509, C44.510, C44.511, C44.519, C44.520, C44.521, C44.529, C44.590, C44.591, C44.599, C44.601, C44.602, C44.609, C44.611, C44.612, C44.619, C44.621, C44.622, C44.629, C44.691, C44.692, C44.699, C44.701, C44.702, C44.709, C44.711, C44.712, C44.719, C44.721, C44.722, C44.729, C44.791, C44.792, C44.799, C44.80, C44.81, C44.82, C44.89, C44.90, C44.91, C44.92, C44.99, C45.0, C45.1, C45.2, C45.7, C45.9, C46.0, C46.1, C46.2, C46.3, C46.4, C46.50, C46.51, C46.52, C46.7, C46.9, C47.0, C47.10, C47.11, C47.12, C47.20, C47.21, C47.22, C47.3, C47.4, C47.5, C47.6, C47.8, C47.9, C48.0, C48.1, C48.2, C48.8, C49.0, C49.10, C49.11, C49.12, C49.20, C49.21, C49.22, C49.3, C49.4, C49.5, C49.6, C49.8, C49.9, C50.011, C50.012, C50.019, C50.021, C50.022, C50.029, C50.111, C50.112, C50.119, C50.121, C50.122, C50.129, C50.211, C50.212, C50.219, C50.221, C50.222, C50.229, C50.311, C50.312, C50.319, C50.321, C50.322, C50.329, C50.411, C50.412, C50.419, C50.421, C50.422, C50.429, C50.511, C50.512, C50.519, C50.521, C50.522, C50.529, C50.611, C50.612, C50.619, C50.621, C50.622, C50.629, C50.811, C50.812, C50.819, C50.821, C50.822, C50.829, C50.911, C50.912, C50.919, C50.921, C50.922, C50.929, C51.0, C51.1, C51.2, C51.8, C51.9, C52, C53.0, C53.1, C53.8, C53.9, C54.0, C54.1, C54.2, C54.3, C54.8, C54.9, C55, C56.1, C56.2, C56.3, C56.9, C57.00, C57.01, C57.02, C57.10, C57.11, C57.12, C57.20, C57.21, C57.22, C57.3, C57.4, C57.7, C57.8, C57.9, C58, C60.0, C60.1, C60.2, C60.8, C60.9, C61, C62.00, C62.01, C62.02, C62.10, C62.11, C62.12, C62.90, C62.91, C62.92, C63.00, C63.01, C63.02, C63.10, C63.11, C63.12, C63.2, C63.7, C63.8, C63.9, C64.1, C64.2, C64.9, C65.1, C65.2, C65.9, C66.1, C66.2, C66.9, C67.0, C67.1, C67.2, C67.3, C67.4, C67.5, C67.6, C67.7, C67.8, C67.9, C68.0, C68.1, C68.8, C68.9, C69.00, C69.01, C69.02, C69.10, C69.11, C69.12, C69.20, C69.21, C69.22, C69.30, C69.31, C69.32, C69.40, C69.41, C69.42, C69.50, C69.51, C69.52, C69.60, C69.61, C69.62, C69.80, C69.81, C69.82, C69.90, C69.91, C69.92, C70.0, C70.1, C70.9, C71.0, C71.1, C71.2, C71.3, C71.4, C71.5, C71.6, C71.7, C71.8, C71.9, C72.0, C72.1, C72.20, C72.21, C72.22, C72.30, C72.31, C72.32, C72.40, C72.41, C72.42, C72.50, C72.59, C72.9, C73, C74.00, C74.01, C74.02, C74.10, C74.11, C74.12, C74.90, C74.91, C74.92, C75.0, C75.1, C75.2, C75.3, C75.4, C75.5, C75.8, C75.9, C76.0, C76.1, C76.2, C76.3, C76.40, C76.41, C76.42, C76.50, C76.51, C76.52, C76.8 |
| Cerebrovascular disease | I60, I60.0, I60.1, I60.2, I60.3, I61, I61.0, I61.1, I61.2, I61.3, I62, I62.0, I62.1, I62.9, I63, I63.0, I63.1, I63.2, I63.3, I63.4, I63.5, I64, I65, I65.0, I65.1, I65.2, I65.8, I65.9, I66.0, I66.1, I66.9, I66, I67, I67.0, I67.1, I67.9, I68, I68.0, I68.1, Z86.73, I69.39, I69.00, I69.10, I69.20, I69.30, I69.40, I69.50, I69.8 |
| Heart failure | I50.1, I50.20, I50.21, I50.22, I50.23, I50.30, I50.31, I50.32, I50.33, I50.40, I50.41, I50.42, I50.43, I50.810, I50.811, I50.812, I50.813, I50.814, I50.82, I50.83, I50.84, I50.89, I50.9 |
| Dementia | F00, F00.0, F00.1, F00.9, F01, F01.0, F01.1, F01.2, F01.3, F01.8, F01.9, F02, F02.0, F02.1, F02.2, F02.3, F02.4, F02.8, F02.9, F03, G30, G30.0, G30.1, G30.9, G31, G31.0, G31.1, G31.8, G31.9 |
| Diabetes | E08.00, E08.01, E08.10, E08.11, E08.21, E08.22, E08.29, E08.311, E08.319, E08.3211, E08.3212, E08.3213, E08.3219, E08.3291, E08.3292, E08.3293, E08.3299, E08.3311, E08.3312, E08.3313, E08.3319, E08.3391, E08.3392, E08.3393, E08.3399, E08.3411, E08.3412, E08.3413, E08.3419, E08.3491, E08.3492, E08.3493, E08.3499, E08.3511, E08.3512, E08.3513, E08.3519, E08.3521, E08.3522, E08.3523, E08.3529, E08.3531, E08.3532, E08.3533, E08.3539, E08.3541, E08.3542, E08.3543, E08.3549, E08.3551, E08.3552, E08.3553, E08.3559, E08.3591, E08.3592, E08.3593, E08.3599, E08.36, E08.37X1, E08.37X2, E08.37X3, E08.37X9, E08.39, E08.40, E08.41, E08.42, E08.43, E08.44, E08.49, E08.51, E08.52, E08.59, E08.610, E08.618, E08.620, E08.621, E08.622, E08.628, E08.630, E08.638, E08.649, E08.65, E08.69, E08.8, E08.9, E09.00, E09.01, E09.10, E09.11, E09.21, E09.22, E09.29, E09.311, E09.319, E09.3211, E09.3212, E09.3213, E09.3219, E09.3291, E09.3292, E09.3293, E09.3299, E09.3311, E09.3312, E09.3313, E09.3319, E09.3391, E09.3392, E09.3393, E09.3399, E09.3411, E09.3412, E09.3413, E09.3419, E09.3491, E09.3492, E09.3493, E09.3499, E09.3511, E09.3512, E09.3513, E09.3519, E09.3521, E09.3522, E09.3523, E09.3529, E09.3531, E09.3532, E09.3533, E09.3539, E09.3541, E09.3542, E09.3543, E09.3549, E09.3551, E09.3552, E09.3553, E09.3559, E09.3591, E09.3592, E09.3593, E09.3599, E09.36, E09.37X1, E09.37X2, E09.37X3, E09.37X9, E09.39, E09.40, E09.41, E09.42, E09.43, E09.44, E09.49, E09.51, E09.52, E09.59, E09.610, E09.618, E09.620, E09.621, E09.622, E09.628, E09.630, E09.638, E09.649, E09.65, E09.69, E09.8, E09.9, E10.10, E10.11, E10.21, E10.22, E10.29, E10.311, E10.319, E10.3211, E10.3212, E10.3213, E10.3219, E10.3291, E10.3292, E10.3293, E10.3299, E10.3311, E10.3312, E10.3313, E10.3319, E10.3391, E10.3392, E10.3393, E10.3399, E10.3411, E10.3412, E10.3413, E10.3419, E10.3491, E10.3492, E10.3493, E10.3499, E10.3511, E10.3512, E10.3513, E10.3519, E10.3521, E10.3522, E10.3523, E10.3529, E10.3531, E10.3532, E10.3533, E10.3539, E10.3541, E10.3542, E10.3543, E10.3549, E10.3551, E10.3552, E10.3553, E10.3559, E10.3591, E10.3592, E10.3593, E10.3599, E10.36, E10.37X1, E10.37X2, E10.37X3, E10.37X9, E10.39, E10.40, E10.41, E10.42, E10.43, E10.44, E10.49, E10.51, E10.52, E10.59, E10.610, E10.618, E10.620, E10.621, E10.622, E10.628, E10.630, E10.638, E10.649, E10.65, E10.69, E10.8, E10.9, E11.00, E11.01, E11.10, E11.11, E11.21, E11.22, E11.29, E11.311, E11.319, E11.3211, E11.3212, E11.3213, E11.3219, E11.3291, E11.3292, E11.3293, E11.3299, E11.3311, E11.3312, E11.3313, E11.3319, E11.3391, E11.3392, E11.3393, E11.3399, E11.3411, E11.3412, E11.3413, E11.3419, E11.3491, E11.3492, E11.3493, E11.3499, E11.3511, E11.3512, E11.3513, E11.3519, E11.3521, E11.3522, E11.3523, E11.3529, E11.3531, E11.3532, E11.3533, E11.3539, E11.3541, E11.3542, E11.3543, E11.3549, E11.3551, E11.3552, E11.3553, E11.3559, E11.3591, E11.3592, E11.3593, E11.3599, E11.36, E11.37X1, E11.37X2, E11.37X3, E11.37X9, E11.39, E11.40, E11.41, E11.42, E11.43, E11.44, E11.49, E11.51, E11.52, E11.59, E11.610, E11.618, E11.620, E11.621, E11.622, E11.628, E11.630, E11.638, E11.649, E11.65, E11.69, E11.8, E11.9, E13.00, E13.01, E13.10, E13.11, E13.21, E13.22, E13.29, E13.311, E13.319, E13.3211, E13.3212, E13.3213, E13.3219, E13.3291, E13.3292, E13.3293, E13.3299, E13.3311, E13.3312, E13.3313, E13.3319, E13.3391, E13.3392, E13.3393, E13.3399, E13.3411, E13.3412, E13.3413, E13.3419, E13.3491, E13.3492, E13.3493, E13.3499, E13.3511, E13.3512, E13.3513, E13.3519, E13.3521, E13.3522, E13.3523, E13.3529, E13.3531, E13.3532, E13.3533, E13.3539, E13.3541, E13.3542, E13.3543, E13.3549, E13.3551, E13.3552, E13.3553, E13.3559, E13.3591, E13.3592, E13.3593, E13.3599, E13.36, E13.37X1, E13.37X2, E13.37X3, E13.37X9, E13.39, E13.40, E13.41, E13.42, E13.43, E13.44, E13.49, E13.51, E13.52, E13.59, E13.610, E13.618, E13.620, E13.621, E13.622, E13.628, E13.630, E13.638, E13.649, E13.65, E13.69, E13.8, E13.9 |
| Drug use disorder | F11.1, F11.10, F11.11, F11.12, F11.13, F11.14, F11.15, F11.16, F11.17, F11.2, F11.20, F11.21, F11.22, F11.23, F11.24, F11.25, F11.26, F11.27, F1, F12, F12.1, F12.10, F12.11, F12.12, F12.13, F12.14, F12.15, F12.16, F12.17, F12.2, F12.20, F12.21, F12.22, F12.23, F12.24, F12.25, F12.26, F12.27, F14, F14.1, F14.10, F14.11, F14.12, F14.13, F14.14, F14.15, F14.16, F14.17, F14.2, F14.20, F14.21, F14.22, F14.23, F14.24, F14.25, F14.26, F14.27, F15, F15.1, F15.10, F15.11, F15.12, F15.13, F15.14, F15.15, F15.16, F15.17, F15.2, F15.20, F15.21, F15.22, F15.23, F15.24, F15.25, F15.26, F15.27, F19, F19.1, F19.10, F19.11, F19.12, F19.13, F19.14, F19.15, F19.16, F19.17, F19.2, F19.20, F19.21, F19.22, F19.23, F19.24, F19.25, F19.26, F19.27 |
| Cocaine use | F14.10, F14.11, F14.120, F14.121, F14.122, F14.129, F14.14, F14.150, F14.151, F14.159, F14.180, F14.181, F14.182, F14.188, F14.19, F14.20, F14.21, F14.220, F14.221, F14.222, F14.229, F14.23, F14.24, F14.250, F14.251, F14.259, F14.280, F14.281, F14.282, F14.288, F14.29, F14.90, F14.91, F14.920, F14.921, F14.922, F14.929, F14.94, F14.950, F14.951, F14.959, F14.980, F14.981, F14.982, F14.988, F14.99 |
| Hypertension | I10, I11.0, I11.9, I12.0, I12.9, I13.0, I13.10, I13.11, I13.2, I15.0, I15.1, I15.2, I15.8, I15.9 |
| Severe liver disease | K70.2, K70.3, K70.40, K70.41, K71.7, K72.00, K72.01, K72.10, K72.11, K72.90, K72.91, K74.0, K74.1, K74.2, K74.60, K74.69, K76.6, K76.7 |
| Morbid obesity | E66.01 |
| Paralysis | G81, G81.0, G81.1, G81.9, G82, G82.0, G82.00, G82.01, G82.02, G82.03, G82.1, G82.10, G82.11, G82.12, G82.13, G83, G83.0, G83.1, G83.2, G83.3, G83.4, G83.8, G83.9 |
| Peripheral vascular disease | I70, I70.0, I70.1, I70.2, I70.3, I70.9, I71, I71.0, I71.1, I71.2, I71.3, I71.4, I72, I72.0, I72.1, I72.2, I72.3, I72.8, I72.9, I73, I73.0, I73.1, I73.9, I74, I74.0, I74.1, I74.2, I74.3, I74.4, I74.8, I74.9, I77, I77.0, I77.1, I77.2, I79, I79.0, I79.1, I79.2, I79.3 |
| Advanced renal failure | N18.5, N18.6 |
| Hypothyroidism | E03, E03.0, E03.1, E03.10, E03.11, E03.2, E03.3, E03.4, E03.5, E03.6, E03.8, E03.9 |
| Valvular Disease | I34, I34.0, I34.1, I34.2, I34.8, I34.9, I35, I35.0, I35.1, I35.2, I35.8, I35.9, I36, I36.0, I36.1, I36.2, I36.8, I36.9, I37, I37.0, I37.1, I37.8, I37.9, I38, I38.0, I38.1, I38.2, I38.3, I38.4, I38.8, I38.9, I39, I39.0, I39.1, I39.2, I39.8, I39.9 |
| Prior cardiac surgery | Z95.1, Z95.2 |
| Methamphetamine use | F15, F15.1, F15.10, F15.11, F15.12, F15.13, F15.14, F15.15, F15.16, F15.17, F15.2, F15.20, F15.21, F15.22, F15.23, F15.24, F15.25, F15.26, F15.27 |
| ACS/MI | I20.0, I21, I21.0, I21.1, I21.9, I21.01, I21.02, I21.03, I21.09, I21.11, I21.12, I21.19, I21.8, I21.9 |
| COPD | J44.0, J44.1, J44.81, J44.89, J44.9 |
| Renal malperfusion | N17.0, N17.1, N17.2, N17.8, N17.9, N28.0 |
| Mesenteric malperfusion | K55.0 |
| Neurologic malperfusion | I63.9, I67.81, I67.82, I67.89, G45.0, G45.1, G45.2, G45.8, G45.9, G46.3, G46.4, G46.5, G46.6, G46.7, G46.8, R29.818 |
| Coronary malperfusion | I20.0, I20.1, I20.8, I20.9, I21.01, I21.02, I21.09, I21.11, I21.19, I21.21, I21.29, I21.3, I21.4, I24.0, I24.8, I24.9, I25.10, I25.110, I25.111, I25.118, I25.119, I25.2, I25.5, I25.6, I25.700, I25.701, I25.708, I25.709, I25.710, I25.711, I25.718, I25.719, I25.720, I25.721, I25.728, I25.729, I25.730, I25.731, I25.738, I25.739, I25.750, I25.751, I25.758, I25.759, I25.760, I25.761, I25.768, I25.769, I25.790, I25.791, I25.798, I25.799 |

Supplemental Table 1: Corresponding ICD-10 Codes for Baseline Characteristics of Patients from Nationwide Readmissions Database.

| Procedure Code | |
| --- | --- |
| 025X0ZZ | Destruction of Thoracic Aorta, Ascending/Arch, Open Approach |
| 02BX0ZX | Excision of Thoracic Aorta, Ascending/Arch, Open Approach, Diagnostic |
| 02BX0ZZ | Excision of Thoracic Aorta, Ascending/Arch, Open Approach |
| 02QX0ZZ | Repair Thoracic Aorta, Ascending/Arch, Open Approach |
| 02RX07Z | Replacement of Thoracic Aorta, Ascending/Arch with Autologous Tissue Substitute, Open Approach |
| 02RX08Z | Replacement of Thoracic Aorta, Ascending/Arch with Zooplastic Tissue, Open Approach |
| 02RX0JZ | Replacement of Thoracic Aorta, Ascending/Arch with Synthetic Substitute, Open Approach |
| 02RX0KZ | Replacement of Thoracic Aorta, Ascending/Arch with Nonautologous Tissue Substitute, Open Approach |
| 02UX07Z | Supplement Thoracic Aorta, Ascending/Arch with Autologous Tissue Substitute, Open Approach |
| 02UX08Z | Supplement Thoracic Aorta, Ascending/Arch with Zooplastic Tissue, Open Approach |
| 02UX0JZ | Supplement Thoracic Aorta, Ascending/Arch with Synthetic Substitute, Open Approach |
| 02UX0KZ | Supplement Thoracic Aorta, Ascending/Arch with Nonautologous Tissue Substitute, Open Approach |
| 02VX0CZ | Restriction of Thoracic Aorta, Ascending/Arch with Extraluminal Device, Open Approach |
| 02VX0DZ | Restriction of Thoracic Aorta, Ascending/Arch with Intraluminal Device, Open Approach |
| 02VX0EZ | Restriction of Thoracic Aorta, Ascending/Arch with Branched or Fenestrated Intraluminal Device, One or Two Arteries, Ope... |
| 02VX0FZ | Restriction of Thoracic Aorta, Ascending/Arch with Branched or Fenestrated Intraluminal Device, Three or More Arteries, ... |
| 02VX0ZZ | Restriction of Thoracic Aorta, Ascending/Arch, Open Approach |

Supplemental Table 2: Procedure Codes Queried in Nationwide Readmissions Database to Identify Surgical Intervention of TAAD. TAAD=Type A aortic dissection
